# Disparities in Mitral Valve Disease Associated with Heart Failure

**DOI:** 10.31083/j.rcm2504129

**Published:** 2024-04-01

**Authors:** Olivia Foley, Rebecca Hammond, Kristine Au, Noureen Asghar, Abubakar Tauseef, Ali Bin Abdul Jabbar, Paul Millner, Mohsin Mirza

**Affiliations:** ^1^Creighton University School of Medicine, Omaha, NE 68178, USA; ^2^Creighton University School of Medicine, Phoenix, AZ 85012, USA; ^3^Internal Medicine, Creighton University Medical Center – Bergan Mercy, Omaha, NE 68124, USA

**Keywords:** epidemiology, disparities, HF, MVD, outcomes, prevalence, treatment

## Abstract

Heart failure (HF) affects millions of people around the world and is a prevalent health issue in the United States. In many cases, HF has an intricate connection with mitral valvular disease (MVD), which can alter a patient’s disease course. Factors such as gender, race, ethnicity, and social determinants of health impact the prevalence, etiology, and treatment of MVD associated with HF. This literature review examines the connection between MVD and HF among adult patients, considering MVD as both a cause and an outcome of HF. This article also identifies the differences in epidemiology and treatment of MVD associated with HF across different gender, ethnicity, race, and socioeconomic groups. This is in an effort to not only identify currently overlooked disparities but to highlight potential ways to improve them. MVD was analyzed based on its hemodynamic subtypes, mitral regurgitation (MR) and mitral stenosis (MS), as these subtypes encompass different etiologies of MVD. The purpose of this article was to identify broad disparities in MVD in association with HF in the adult population. The results of this study found stark differences between prevalence, treatment, and disease outcomes across groups. Women and Black patients were identified as high-risk for under-utilization and prescription delay of treatment options. Women were often treated at more advanced stages of MVD, while treatment was often delayed in Black patient populations. Factors such as these impact treatment outcomes. Conversely, men and White patients were identified as lower-risk groups for treatment inadequacies and poor HF and MVD related outcomes. Socioeconomic status (SES) was also found to play a role, with low SES being a risk factor for developing rheumatic heart disease. Low SES groups are also more likely to develop HF, which predisposes to secondary MR. Despite general knowledge of these disparities, few studies analyze HF and MVD for specific groups. This literature review is thus necessary to identify current inequities in care and underscore potential solutions to raise awareness for further research efforts and funding. This analysis identifies MVD treatment guidelines and contributing social determinants of health as areas that must be addressed to minimize HF and MVD disparities.

## 1. Introduction

In the United States, 15% of all deaths due to heart valvular disease involve the mitral valve [1]. Mitral valvular disease (MVD) encompasses multiple different disease phenotypes and is both a cause and consequence of heart failure (HF) [2, 3]. Broadly, MVD can be subclassified as either mitral regurgitation (MR) or mitral stenosis (MS). Both MR and MS are causes of HF, with MR also being a sequela of HF.

Approximately 5 million people in the United States are affected by MR, which is expected to rise in future years [2]. MR is classified as either a primary or secondary disease [2]. Primary disease has structural origins intrinsic to the valve, commonly caused by mitral valve prolapse (MVP), with MVP being a common valvular disease in the United States and worldwide [2, 4, 5]. Secondary MR is a more common cause of severe MR and results from left ventricular dysfunction and pre-existing myocardial disease [5]. The presence of severe MR due to left ventricular dysfunction is a poor prognostic sign in HF [2]. MR is also an independent risk factor for decreased survival in patients with left ventricular dysfunction [6]. Risk factors for secondary MR parallel risk factors for left ventricular dysfunction, including coronary artery disease and HF [5].

MS can be categorized as rheumatic or non-rheumatic in origin. The major cause of MS is rheumatic heart disease after prior infection with group A streptococcus [3]. Overall, the incidence of rheumatic MS in developed countries is 1/100,000, making it less common than MR [3]. Though there is limited data on the prevalence of non-rheumatic causes of MS, one study found that 18.5% of their patients with MS had non-rheumatic stenosis which is primarily caused by mitral annulus calcification [7]. The majority of patients with MS will not survive more than 10 years after symptom onset, with HF being a common outcome of advanced MS [3]. Given that both MR and MS cause significant morbidity and mortality, especially when concurrent with HF, it is vital to understand and address these disparities to provide quality care to the patients.

## 2. Materials and Methods

It is well established that socioeconomic, gender, racial, and ethnic disparities exist in the diagnosis and treatment of cardiovascular disease (CVD). This study sought to review these disparities in the context of MVD among adult patients and its relationship to HF. MVD is unique in that the etiology, prevalence, and treatment of the different subtypes vary widely, yet all are causes or outcomes of HF. In this paper, any relevant articles addressing disparities in etiology, prevalence, and treatment of the different types of MVD and MVD related HF were reviewed. We also analyzed data from the Centers for Disease Control and Prevention WONDER (Wide-Ranging Online Data for Epidemiologic Research) Multiple Cause of Death dataset from 1999 to 2020. All deaths related to MR and HF and MS and HF in patients 15 years and older were included. Data for deaths related to MR and HF were obtained using International Classification of Diseases- 10th Revision codes I50 (Heart failure), I50.1 (Rheumatic mitral insufficiency), and I34.0 (Mitral (valve) insufficiency) and were queried together to ascertain patients who had MR and HF as underlying causes of death. Data for deaths related to MS and HF were obtained using codes I50 (Heart failure), I05.0 (Mitral stenosis), and I34.2 (Nonrheumatic mitral (valve) stenosis) and were similarly queried together to ascertain patients who had both MS and HF as underlying causes of death. Sociodemographic data across the United States was acquired for both patient subgroups. Total deaths were extracted.

## 3. Literature Review & Results

Social determinants of health impact the development of MVD and HF among different groups. One of the predominant contributing factors is socioeconomic status (SES). In the United States, rheumatic fever, a precursor to rheumatic heart disease, and rheumatic heart disease prevalence remains higher in low-income populations than in high-income populations [4, 5]. Some risk factors for developing rheumatic heart disease are socioeconomic, including overcrowding, poor health infrastructure, and lack of access to a healthcare system [5, 8]. While to our knowledge there is little data regarding the relationship of SES and primary MR, it is established that low SES in developed countries is an independent risk factor for HF which predisposes to secondary MR [9].

**Fig. 1.**
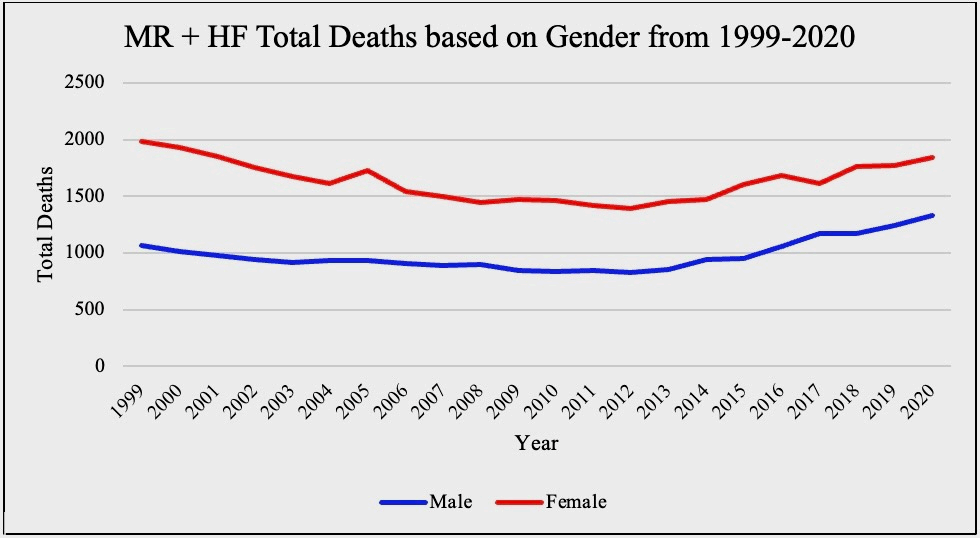
**Mitral regurgitation (MR) and heart failure (HF) total death based on gender**. Results of a CDC WONDER database review comparing total death in female vs. male patients 15 years or older with MR and HF as either their primary or secondary diagnosis. CDC WONDER, Center for Disease Control and Prevention Wide-ranging Online Data for Epidemiologic Research.

**Fig. 2.**
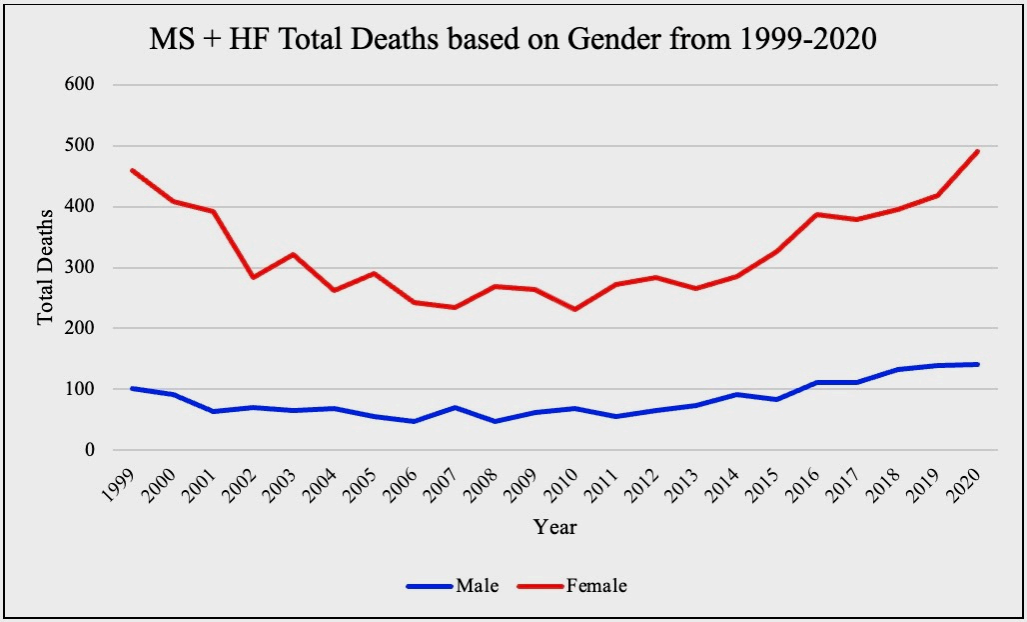
**Mitral stenosis (MS) and heart failure (HF) total deaths based on gender**. Results of a CDC WONDER database review comparing total death in female vs. male patients 15 years or older with MS and HF as either their primary or secondary diagnosis. CDC WONDER, Center for Disease Control and Prevention Wide-ranging Online Data for Epidemiologic Research.

MVD prevalence also varies widely among different populations. While MR is equally prevalent between men and women, the etiology of MR varies between them. MVP is more common among women than men; thus, women are more likely to be affected by primary MR, while men are more likely to be affected by secondary MR [10]. Further, a thick, myxomatous valve is more common in women than in men [10]. The valvular abnormalities that are more common in women are considered more surgically complex. Women are also at higher risk for developing MR after other cardiac complications, such as myocardial infarction or coronary artery disease [10]. There are higher rates of mitral annulus calcification, a cause of degenerative MS, in women compared to men which can also complicate MR [11]. When comparing incidence of rheumatic MS among men and women, women who have gone through puberty have an increased incidence of this disease [5]. Our analysis of the Center for Disease Control and Prevention Wide-ranging Online Data for Epidemiologic Research (CDC WONDER) data also shows that total deaths from both MR and MS in association with HF are substantially higher in females versus males overall. This information is summarized in Figs. 1,2 below.

MVD prevalence also varies amongst racial and ethnic groups. While MVP is more common in White patients, Black patients are more likely to have secondary MR than White and Asian populations [12]. In a study of approximately 1400 patients who underwent mitral valve interventions, authors found that Black patients had a significantly higher incidence of rheumatic MVD [13]. Further, in a review of 9000 mitral valve operations across the state of Michigan, Black patients again had a higher prevalence of rheumatic MVD [14]. These studies suggest an increased risk of rheumatic MVD in Black patients compared to White patients. Black individuals are at the highest risk of developing HF, which predisposes to secondary MR, followed by Hispanic, White, and Asian populations. Black and Hispanic populations also develop HF at a younger age and have higher hospitalization rates [15].

Disparities exist in the treatment of MR and MS, though the guidelines for treating MR and MS are different. For MS, the definitive therapy is mechanical relief of the stenotic valve [16]. For primary MR, treatment consists of valve repair or replacement once the patient reaches a certain level of symptomatology or degree of dysfunction [17]. Finally, for secondary MR associated with HF, guideline-directed medical therapy (GDMT) is first-line treatment for both HF and resultant MR, followed by cardiac resynchronization therapy (CRT) and transcatheter and surgical valvular interventions [18]. While medicine has made great strides in advancing treatment for MVD and associated HF, the benefits of this progression have not reached all communities equally.

When analyzing treatment availability based on gender, women are less likely than men to undergo surgery to correct MVD and are often not offered this treatment until their disease is at a more advanced stage [12]. When looking specifically at MR, men are more likely to receive surgical intervention than women. This is due to multiple factors. First, women generally present with more complex valvular disease and are more likely to have mitral annular calcification in conjunction with MR. Additionally, women are less likely to meet the surgical criteria of enlarged left ventricular and left atrial size despite the same severity of regurgitation as men [19, 20]. This is primarily due to their lack of representation in initial studies of MR, with only men being utilized to set cut-off values for the cardiac dimension limits that necessitate surgical repair. Inequities such as this contribute to the gender disparity seen in outcomes, with women being referred for interventions at older ages or at later stages of HF [10, 12]. Despite mitral valve repair being preferred over replacement due to lower complication rates, women are more likely to be treated with replacement given their complex valvular disease, leading to worse outcomes [10]. Non-surgical options for treating secondary MR and HF, such as CRT, are also under-utilized in women, highlighting concerning treatment disparities between the genders overall [21]. Despite these treatment inequities, women are seen to have favorable outcomes in certain instances. When women are treated with percutaneous balloon valvuloplasty for rheumatic MS, they tend to have favorable outcomes. Degenerative MS is more common in women, however, and can complicate treatment [19].

Treatment disparities based on race and ethnicity are also prolific, with under-prescription of GDMT, the primary treatment option for secondary MR and HF, in Black patients [12, 22]. Black patients are also significantly less likely to receive CRT than White patients [21]. CRT-D, a combination of CRT and implantable cardioverter defibrillator (ICD), has been shown in multiple studies to be underutilized in both Black and Hispanic patients [23, 24]. As interventional options have improved, there has been uneven distribution amongst HF patients, with the proportion of Black patients who receive percutaneous MV interventions remaining stagnant, while the number of White patients receiving this option has grown substantially [22]. Studies have found that Black patients are less likely to undergo mitral valvuloplasty, especially when their disease is non-rheumatic in origin [13]. Hispanic are also less likely to receive mitral valve interventions, specifically Transcatheter Mitral Valve Replacement (TMVR), an important technique for patients at high open surgical risk [25]. Transcatheter Edge to Edge Repair (TEER) is a similarly important technique for high-risk patients. In a review of TEER procedures from State Inpatient Databases, Black and Hispanic patients were found to lack access to high volume TEER centers in comparison to White patients. Concerningly, Hispanic patients also experienced increased mortality [26]. Additionally, studies have found that Black patients with MVD present younger with increased comorbidities, including HF, and experience a higher frequency of postoperative complications [13, 14].

## 4. Discussion

Disparities in MVD associated with HF, particularly in females and racial and ethnic minority populations, pose significant challenges in achieving equitable and optimal care for all patients. Despite advancements in treatment options and overall survival rates, these disparities persist or worsen, leading to disproportionate burden and outcomes for certain communities, as summarized in Table 1 (Ref. [4, 5, 8, 9, 10, 11, 12, 13, 14, 19]) and Table 2 (Ref. [12, 13, 19, 20, 21, 22, 23, 24, 25, 26]) below.

**Table 1.** **Disparities in prevalence and etiology of mitral valve disease**.

Factor	Description	Disparity
Mitral regurgitation		
Gender	Male vs. Female	Women are more likely to have primary MR and men are more likely to have secondary MR [10]
		Women are more likely to have surgically complex valvular lesions including concomitant mitral annulus calcification [10, 11, 19]
Race	Black vs. White	Black patients are more likely to have secondary MR [12]
		White patients are more likely to have MVP [13, 14]
		Black patients may be more likely to have rheumatic MVD [13, 14]
		Black patients with MVD present younger with increased comorbidities [13, 14]
SES	Low vs. High	Low SES is a risk factor for developing HF which predisposes to secondary MR [9]
Mitral stenosis		
Gender	Male vs. Female	Women are more likely to develop rheumatic MS [11, 19]
		Women are at increased risk of degenerative MS including mitral annulus calcification [11, 19]
Race	Black vs. White	Black patients are more likely to have rheumatic MVD [13, 14]
		Black patients with MVD present younger with increased comorbidities [13, 14]
SES	Low vs. High	Rates of rheumatic MS are higher in low-income populations [4, 5, 8]

Disparities in MR based on gender, race, and SES and disparities in MS based on gender, race, and SES. HF, heart failure; MR, mitral regurgitation; MS, mitral stenosis; MVD, mitral valvular disease; MVP, mitral valve prolapse; SES, socioeconomic status.

**Table 2.** **Treatment disparities in mitral valve disease**.

Treatment	Disparity
GDMT	Under-prescription in Blacks [12, 22]
Surgical intervention	Women are less likely to receive surgical intervention [19, 20]
	Black patients are less likely to receive mitral valvuloplasty [13]
	Hispanic patients are less likely to receive TMVR [25]
	Decreased access to high volume TEER centers for Hispanic and Black patients [26]
Advanced non-surgical options	CRT is underutilized in women [21]
	CRT is underutilized in Black patients [21, 23, 24]
	CRT is underutilized in Hispanic patients [23, 24]

Disparities in treatment of MVD based on sex, race, and ethnicity. GDMT, guideline-directed medical therapy; CRT, cardiac resynchronization therapy; TEER, transcatheter edge to edge repair; TMVR, transcatheter mitral valve replacement; MVD, mitral valvular disease.

Due to the pervasiveness of MVD and the disparities in prevalence, management, and outcomes among various groups, it is crucial that the underlying inequities be addressed. Revamping the referral system for diverse HF patients with MVD will show immense benefits [22]. For example, women referred for mitral valve surgery should have their cardiac dimensions adjusted for body surface area to ensure correct disease staging [20]. In addition, given that non-White patients are presenting with more severe disease at younger ages, physicians must screen all patients effectively for both rheumatic and degenerative conditions and refer patients to cardiology at earlier stages [13, 14]. Diversifying clinical trial populations would also help expand knowledge of disease courses overall as differences in representation in clinical trials, the subjects of whom have been predominantly White males throughout history, have also contributed to the stark disparities in HF outcomes between groups [12]. Black patients are historically underrepresented in HF clinical trials, and women with valvular heart disease are also underrepresented, resulting in ineffective surgery referral parameters and subsequently worsened outcomes [22, 27]. There is also a lack of studies and categorization of disease characteristics among diverse groups due to society’s tendency to group broad and diverse populations into generic racial categories. This prevents in-depth studies of the health and outcomes of specific racial and ethnic groups, leading to a lack of accurate data and worse outcomes overall [15].

Social determinants of health play a significant role in an individual’s health and overall well-being, with SES shown to significantly impact CVD overall [28]. Given that SES is a risk factor for the development of MS and HF and subsequently secondary MR, solutions must include increasing access to health screening programs that allow for early identification of MVD. Social support and economic stability are key in producing good health outcomes, particularly with CVD; thus, these facets need to be incorporated into broad programs to correct HF disparities moving forward [28]. Potential solutions to these problems would involve evidence-based intervention programs involving multi-stakeholder engagement to target every aspect of life and health that plays a role in CVD [22]. Along with this, distribution of accessible cardiologists must be improved by implementing interventions such as physician incentives and telemedicine [12]. It is also important to specifically increase access to high volume centers for transcatheter and surgical interventions as volume is associated with outcomes for both treatments [26, 29].

## 5. Conclusions

By implementing these strategies and working collectively to improve the health and longevity of patients with HF and valvular heart disease, we can strive towards a more equitable and effective healthcare system for all individuals, regardless of SES, gender, race, or ethnicity.

Potential limitations of this literature review include the possibility of missing relevant studies despite comprehensive search strategies. There also may be gaps in specific data related to gender-based incidence and mortality rates in MR due to limited large-scale population studies focused specifically on these factors. Current available data tends to focus on overall MR trends and outcomes, irrespective of disparities. This not only highlights the need for future study but also a potential avenue of research, working to improve the database on gender and race related disparities in different forms of MVD overall. Another limitation of the study includes underestimation of MVD prevalence given the potential limitations of diagnostic techniques used to diagnose valvular abnormalities. Therefore, the relationship between MVD and HF may be incompletely characterized by present data. Additionally, given that MVD is a heterogenous entity, differences in the prevalence of MVD and HF described in this article could be in part due to differences in the classification of MVD.

## Abbreviations

CDC WONDER, Center for Disease Control and Prevention Wide-ranging Online Data for Epidemiologic Research; CRT, cardiac resynchronization therapies; CVD, cardiovascular disease; GDMT, guideline-directed medical therapy; HF, heart failure; ICD, implanted cardioverter defibrillators; MR, mitral regurgitation; MS, mitral stenosis; MVD, mitral valvular disease; MVP, mitral valve prolapse; SES, socioeconomic status; TEER, transcatheter edge to edge repair; TMVR, transcatheter mitral valve replacement.

## Supplementary Material

Supplementary material associated with this article can be found, in the online version, at https://doi.org/10.31083/j.rcm2504129.
