# Supplementary material for: Disparities in Mitral Valve Disease Associated with Heart Failure
Source: Rev Cardiovasc Med. 2024 Apr 1;25(4):129. doi: 10.31083/j.rcm2504129 (PMC11264015; doi:10.31083/j.rcm2504129)
Supplement: Supplementary file 1 [file 2153-8174-25-4-129-s1.pdf]

MR + HF Total Deaths based on Gender:

Year	Male	Female
1999	1070	1981
2000	1016	1931
2001	978	1848
2002	941	1750
2003	919	1674
2004	932	1609
2005	932	1731
2006	911	1546
2007	892	1494
2008	899	1444
2009	844	1474
2010	841	1461
2011	849	1416
2012	826	1389
2013	850	1454
2014	946	1470
2015	952	1605
2016	1059	1681
2017	1170	1615
2018	1170	1762
2019	1244	1772
2020	1330	1838
Total	21571	35945

<u>MS + HF Total Deaths based on Gender:</u>

Year	Male	Female
1999	101	460
2000	92	408
2001	63	392
2002	71	284
2003	65	322
2004	68	263
2005	55	291
2006	47	243
2007	70	235
2008	47	269
2009	62	264
2010	69	231
2011	56	272
2012	65	283
2013	74	265
2014	91	285
2015	83	326
2016	112	387
2017	111	379
2018	132	396
2019	140	419
2020	141	490
Total:	1815	7164
